# Nerve growth factor: from the early discoveries to the potential clinical use

**DOI:** 10.1186/1479-5876-10-239

**Published:** 2012-11-29

**Authors:** Luigi Aloe, Maria Luisa Rocco, Patrizia Bianchi, Luigi Manni

**Affiliations:** 1 Cellular Biology and Neurobiology Institute, CNR, via del Fosso di Fiorano 64, 00143, Rome, Italy; 2 Institute of Translational Pharmacology, National Research Council (CNR), via Fosso del Cavaliere 100, 00136, Rome, Italy

**Keywords:** Nerve growth factor, Alzheimer’s disease, Parkinson’s disease, Peripheral neuropathies, Skin ulcers, Neurotrophic keratitis, Glaucoma, Hypoxic-ischemic brain injury, Optic glioma

## Abstract

The physiological role of the neurotrophin nerve growth factor (NGF) has been characterized, since its discovery in the 1950s, first in the sensory and autonomic nervous system, then in central nervous, endocrine and immune systems. NGF plays its trophic role both during development and in adulthood, ensuring the maintenance of phenotypic and functional characteristic of several populations of neurons as well as immune cells. From a translational standpoint, the action of NGF on cholinergic neurons of the basal forebrain and on sensory neurons in dorsal root ganglia first gained researcher’s attention, in view of possible clinical use in Alzheimer’s disease patients and in peripheral neuropathies respectively. The translational and clinical research on NGF have, since then, enlarged the spectrum of diseases that could benefit from NGF treatment, at the same time highlighting possible limitations in the use of the neurotrophin as a drug. In this review we give a comprehensive account for almost all of the clinical trials attempted until now by using NGF. A perspective on future development for translational research on NGF is also discussed, in view of recent proposals for innovative delivery strategies and/or for additional pathologies to be treated, such as ocular and skin diseases, gliomas, traumatic brain injuries, vascular and immune diseases.

## Nerve growth factor

**Figure 1:**
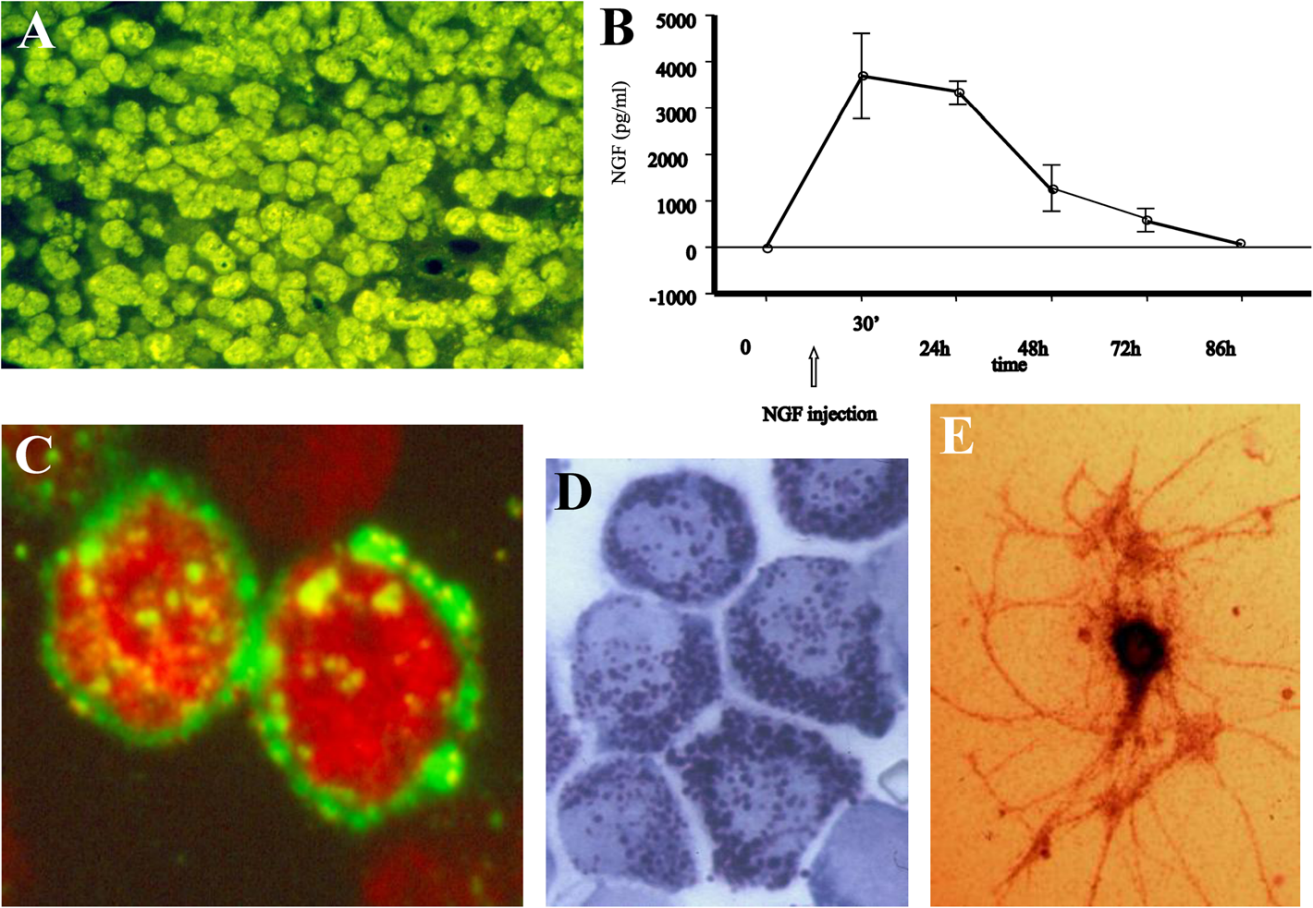
**NGF is produced by every peripheral tissue/organ that is innervated by sensory afferents and/or sympathetic efferents, as well as by central and peripheral nervous system and immune cells.** The largest amount of the neurotrophin is produced in mice submaxillary glands, as revealed by immunofluorescence staining depicted in panel **A**, that are the source for murine NGF used in several clinical trials. When intravenously injected in rats (**B**), NGF levels quickly increases in the bloodstream, reaching a peak within 30 min and remaining above baseline levels up until 72 h. Peripheral NGF injection induces peculiar effects on immune circulating cells, such as the overexpression of its receptor TrkA on circulating lymphocytes (**C**) or degranulation of peritoneal mast cells (**D**). Radiolabelled, intra-cerebroventricular injected NGF is captured by TrkA-expressing neurons, such as cholinergic neurons in the basal forebrain complex (**E**).

Nerve growth factor (NGF) is the first discovered member of the neurotrophin family [1]. NGF is essential for the development and phenotypic maintenance of neurons in the peripheral nervous system (PNS) and for the functional integrity of cholinergic neurons in the central nervous system (CNS) (Figure 1) [2]. The amino acid and messenger RNA sequences of this neurotrophin have been classified and indicate that NGF is a highly conserved molecule that shares considerable homology within different species [3]. The mature, active form of NGF descend from proteolitic cleavage of a precursor form (ProNGF), that have important roles during development and in adult life, having both pro-apoptotic and neurotrophic properties [4,5].

NGF exerts its biological action by challenging the specific receptor tropomyosin kinase receptor A (TrkA), which is a typical tyrosine kinase receptor [6]. The major cytosolic/endosomal pathways activated by the TrkA are Ras-mitogen activated protein kinase (MAPK), extracellular signal-regulated kinase (ERK), phosphatidylinositol 3-kinase (PI3K) -Akt, and Phospholipase C (PLC) -γ [7-9]. NGF also binds to and activate the low-affinity, non-selective p75 pan-neurotrophin receptor (p75^NTR^). This receptor is a transmembrane glycoprotein that regulates signaling through TrkA [9-11]; binding of NGF to p75^NTR^ activates additional signaling pathways that, in the absence of co-expressed TrkA, may signal a cell to die via apoptosis [10-12]. Signaling pathways activated by p75^NTR^ are the Jun kinase signaling cascade, NF-κB and ceramide generation [13].

**Figure 2:**
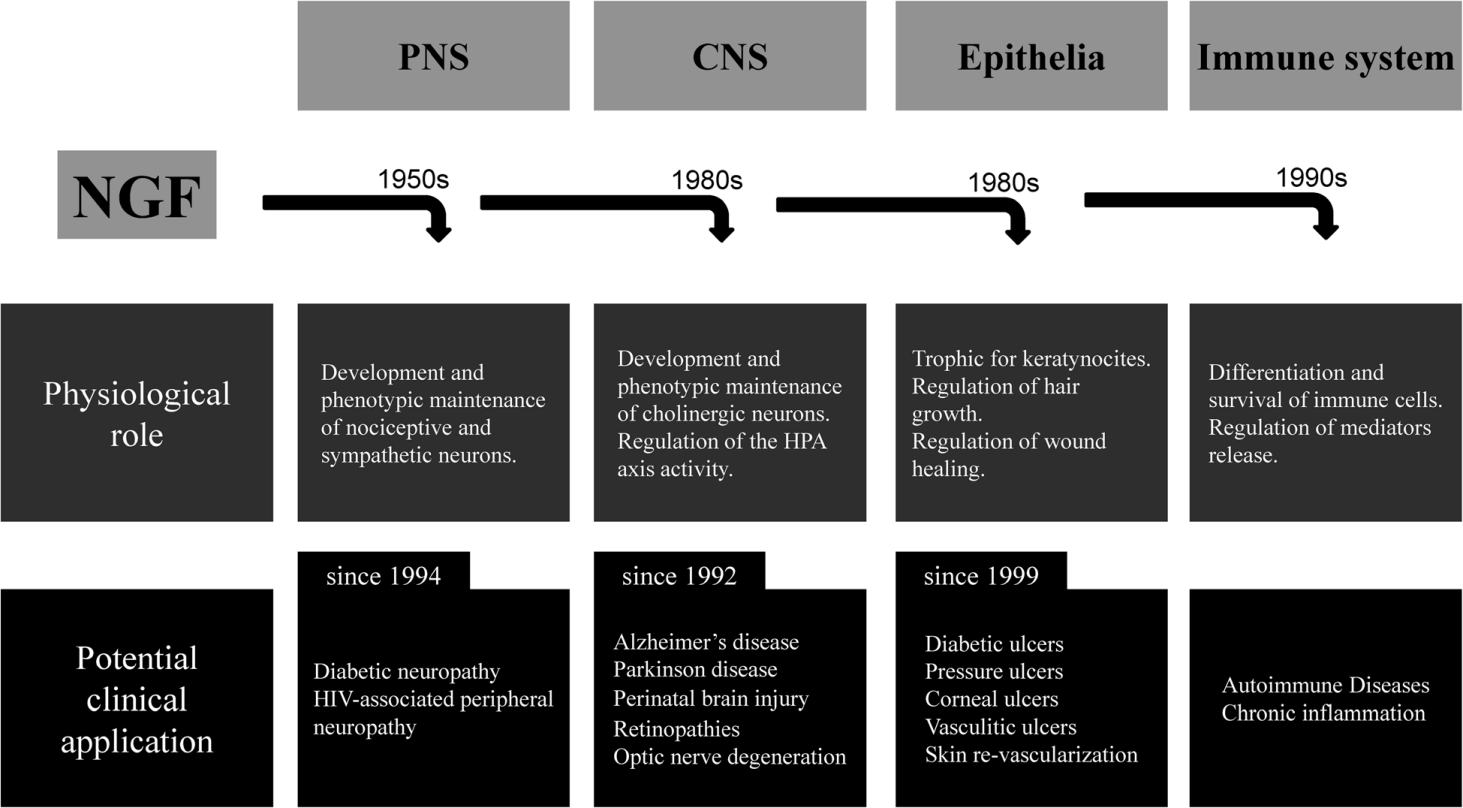
**The huge amount of research data produced since its discovery in the 1950s, first characterized the physiological role of the neurotrophin NGF in the regulation of development and phenotypic maintenance of peripheral nervous system (PNS).** A similar role for central cholinergic neurons was described starting from the 1980s, while more recently NGF has been characterized as a survival, differentiative and trophic factors also for cells belonging to the immune system and the epithelial lineage. Basic and translational research based on such described NGF activities have then explored the possibility to develop NGF-based pharmacotherapies for peripheral neuropathies, brain degenerative and traumatic diseases, several kinds of epithelial derangements. A possible, yet unexplored field for clinical development of NGF as a drug, is based on its activity as immune-regulator, possibly involved in autoimmune and chronic inflammatory pathologies.

The discovery of NGF dated the ‘50s of the last century and was awarded with the Nobel prize in 1986 [13]. In 1953, Rita Levi-Montalcini, working in the Victor Hamburger laboratory at Washington University (Saint Louis, MA, USA), grafted a piece of mouse sarcoma tissue onto chick embryos whose wing buds had been extirpated. She discovered that the tumor tissue produced a soluble factor that promoted the growth of nearby sensory and sympathetic ganglia [13]. Collaborating with the biochemist Stanley Cohen, they isolated the substance responsible and named it NGF. For over 35 years, NGF has been considered as a very powerful and selective growth factor for sympathetic and sensory neurons and for cells derived from the neuronal crest (Figure 2) [14-16]. In these neurons, NGF dynamically controls neurotransmitters and neuropeptides synthesis. In sympathetic neurons the production of norepinephrine is regulated by NGF through selective induction of tyrosine hydroxylase (TH) [17]. In the dorsal root ganglion (DRG) the expression of neuropeptides such as Substance P (SP) and Calcitonin Gene-Related Peptide (CGRP) by primary sensory neurons is under NGF control [18] and in vivo deprivation of NGF, as a result of nerve transection or anti-NGF treatment, causes a marked decrease in SP and CGRP synthesis [19]. NGF supply from the innervation field influences the neuronal plasticity that allows the adult nervous system to modify its structure and functions in response to stimuli. Indeed, the constitutive synthesis of NGF in adult tissues correlates with PNS neurons phenotypic features, such as innervation density, cell body size, axonal terminal sprouting, dendrites arborization, induction or inhibition of neuropeptides and neurotransmitters or transmitter-producing enzymes [17-21].

In the central nervous system (CNS), the greatest amount of NGF is produced in the cortex, the hippocampus and in the pituitary gland; although significant quantities of this neurotrophin are also produced in other areas, including the basal ganglia, thalamus, spinal cord and in the retina [22]. The NGF plays a pivotal role in the survival and function of cholinergic neurons of the basal forebrain complex (BFC) (Figures 1 and 2) [23], such functions include attention, arousal, motivation, memory and consciousness. Since BFC neurons are highly affected in Alzheimer’s disease (AD), NGF has been indicated as a potential protective and/or curative factor for neurodegenerative disorders associated with these neurons [24]. In the CNS, NGF also regulates phenotypic features in noradrenergic nuclei of hypothalamus and brainstem, participating in the central regulation of autonomic response and in the modulation of stress axis activity [25-29].

Cells of the immune-hematopoietic system also produce and utilize NGF [2,30,31]. Since the early description of the effects of NGF on mast cells (Figures 1 and 2) [32,33], the role played by the neurotrophin in the regulation of immune functions and immune cells' behavior has been greatly characterized. NGF receptors are expressed in immune organs and on immune cell populations (Figure 1) allowing NGF to modulate cell differentiation and regulate the immune response. NGF affects the survival and/or differentiation and/or phenotypic features of hematopoietic stem cells [34-37], granulocytes [38-46], lymphocytes [47-54] and monocytes [47,54-59]. NGF concentrations in the tissues change during inflammation and inflammatory mediators induce NGF synthesis in a variety of cell types [60-62]. Enhanced production of NGF has been reported in inflamed tissues of patients with inflammatory and autoimmune diseases [60,61,63], but the reasons why NGF concentration is enhanced and how this can affect inflammatory responses are far from being fully understood.

## Clinical use of NGF

### Peripheral neuropathies

According to the classical neurotrophic model, NGF is produced in and released by target tissues, is then captured by specific receptors expressed on nerve terminals and retrogradely transported to the neuron body, where it exerts its neurotrophic activity [64,65]. Any perturbation in the neurotrophic circuit could generate peripheral nerve dysfunction and neuronal sufferance, as those characterizing peripheral neuropathies. Data obtained in animal models and in human pathologies demonstrated that disease-associated peripheral neuropathies could be associated with either deregulation of NGF synthesis, transport and utilization by PNS neurons [66-71]. This gives to NGF an etiological value in the development of neuropathic symptoms associated with, i.e., diabetes, HIV infections or chemotherapy, and point to the neurotrophin as a possible pharmacological tool in the treatment of peripheral neuropathies (see Table 1 for a comprehensive summary of clinical trials on NGF in peripheral neuropathies).

**Table 1:** Summary of clinical trials with NGF on peripheral neuropathies

**Disease**	**Study type**	**NGF type and dosage**	**Delivery route**	**Outcome**	**Side effects**	**References**
Healthy subjects	Phase I double-masked, randomized, placebo-controlled study.	Recombinant human NGF. Doses ranging from 0.03 to 1 μg/kg.	Subcutaneous, intravenous.	The study evaluated the safety of single doses of rhNGF in healthy human volunteers. No life-threatening adverse events were seen at any dose. Dose-dependent mild to moderate muscle pain and hyperalgesia at the injection site was reported.		[84,85]
Diabetic polyneuropathy	Phase II, placebo-controlled clinical trial.	Recombinant human NGF. 0.1 and 0.3 μg/kg.	Subcutaneous.	Significant improvement of neuropathic symptoms after 6 months of treatment.	Dose-dependent hyperalgesia at the injection site.	[86,87]
	Phase III, randomized, double-blind, placebo-controlled clinical trial.	Recombinant human NGF. 0.1 μg/kg.	Subcutaneous.	Not significant compared to placebo.	Dose-dependent hyperalgesia at the injection site.	[87,88]
HIV-associated peripheral neuropathy	Phase II, multicenter, placebo-controlled, randomized clinical trial.	Recombinant human NGF. 0.1 and 0.3 μg/kg.	Subcutaneous.	Significant improvements in daily and global pain assessments.	Injection site pain. Severe transient myalgic pain.	[92,93]
	Long term (48 weeks) phase II, multicenter, placebo-controlled, randomized clinical trial.	Recombinant human NGF. 0.1 and 0.3 μg/kg.	Subcutaneous.	No improvement in neuropathy severity.	Injection site pain.	[94]

#### Diabetes

Diabetes is often characterized by major complications such as dysfunction and degeneration of several types of PNS neurons/fibers [72,73]. Sensory involvement is predominant, the small diameter sensory fiber degeneration being responsible for the more debilitating symptoms. Deficits of NGF transport [66,67,74], serum and tissue content [66,67,75,76] have been demonstrated in experimental diabetes. Major components of the NGF signaling pathway have been also found deregulated in experimental diabetes [77-79], as well as the production of neuromodulators that is known to be under NGF control [80,81]. On the other hand NGF supply in animal models of diabetic neuropathies reverses neuropathic signs, by protecting the affected PNS neurons and normalizing their activity [82,83].

The production of recombinant human NGF (rhNGF) has been first developed and tested in phase I clinical trial, where moderate side effects, such as myalgias and injection site hyperalgesia, were evidenced in healthy subjects [84,85]. A phase II clinical trial on 250 patients affected by diabetic polyneuropathy was then performed [86,87]. The study revealed a significant improvement of neuropathic symptoms in the NGF-treated patients, but also evidenced the occurrence of side effects, such as injection site hyperalgesia, myalgias and arthralgias, that limited the blinding of the study [86,87]. The results of the study were, however, considered encouraging and clinical trial continued in a phase III study [88]. Those enrolled 1019 patients who were treated subcutaneously with rhNGF, 3 times a week for 48 weeks. The study revealed almost the same side effects of the previous ones, but failed to demonstrate substantial benefits from the NGF treatment. The relative failure of the study, compared to previous phase II trial, was attributed to the low dosage, limited by the occurrence of side effects, but other causes, as the characteristic of patient population, choice of end points, measurement of neuropathy and the possible low quality of the rhNGF used, were not excluded [87].

#### Human immunodeficiency virus

Peripheral nerve complications in human immunodeficiency virus (HIV) patients could arise from the virus itself or from the anti-viral drugs [89-91]. A phase II, multicenter, placebo-controlled, randomized clinical trial with subcutaneous rhNGF on 270 HIV-infected patients affected by sensory neuropathy has been performed a decade ago [92,93]. A significant positive effect of rhNGF was found on neuropathic pain, with injection site pain as the most frequent side effect. It was concluded that rhNGF was safe and well tolerated. In contrast with the latter study, the same group published a report on the long-term (48 weeks) effect of rhNGF in an open-label study of 200 subjects with HIV-associated distal sensory neuropathy [94]. While apparently safe and well tolerated, the NGF did not improve the severity of neuropathy, measured by neurological examination, quantitative sensory testing and epidermal nerve fiber density.

#### Other peripheral neuropathies that could benefit from NGF treatment

In vitro and in vivo studies provided support for clinical trials on rhNGF in chemotherapy-induced peripheral neurotoxicity (CIPN). The development of sensory neuropathies often limits the dosage and time-extension of anti-tumor therapies based on cytotoxic agents [90,95]. NGF has been demonstrated to counteract the reduction of neurite outgrowth from rat DRG in vitro, induced by cisplatin, vincristine or Taxol [96] and the development of behavioral manifestations of cisplatin-induced neuropathy [69,97-101]. Moreover, a positive correlation was found between the decrease of circulating NGF and the severity of CIPN in humans [102].

A decrease in NGF has been also reported in leprosy-affected human skin and nerve [103,104]. The NGF produced by keratinocytes has been found decreased in skin biopsies from leprosy patients [103]. Moreover, a significant loss of intra-epidermal innervation [105], and a lowered expression of sensory neuromodulators that are under NGF control, such as Substance P [104] and the sodium channel SNS/PN3 [103], have been found in leprosy-affected skin. From a clinical standpoint all these NGF system alterations found in leprosy skin correlated with the characteristic sensory deficit and the loss of skin neurotrophism that might lead to trophic ulcers and mutilation [106].

### CNS diseases

Studies on rodents and primates have demonstrated that exogenous NGF was able at neuro-protecting BFC neurons by both traumatic insults and age-related cholinergic decline [107-111]. It has also been demonstrated that NGF could directly act on two classical hallmark of AD: β-amyloid neurotoxicity and tau hyperphosphorylation. Indeed in vitro and in vivo experiments indicated NGF as a direct anti-amyloidogenic factor, being able to regulate both amyloid gene expression and protein processing [112-114]. Furthermore NGF has been shown to counteract tau hyperphosphorylation both in vitro [115] and in vivo [116]. Further studies on human tissues failed to demonstrate a reduction of NGF production in the cortex and hippocampus of AD patients, while the evidence for a decreased NGF immunoreactivity in the BFC suggested that impaired NGF supply via retrograde transport could be the effective cause of cholinergic neurodegeneration in AD [117]. Thus, the correct therapeutic strategy should pursue NGF deliverance in the proximity of cholinergic cell bodies, where the effective NGF deficits have been revealed, rather than in axon terminal regions (i.e. cortex and hippocampus).

The greatest challenge in the delivery of NGF to CNS resides in its inability in crossing the blood–brain barrier (BBB), when systemically administered [118]. For this reason, the intra-cerebro-ventricular (ICV) way of NGF delivery has been pursued in AD patients in two separate clinical trials (see Table 2 for a summary of NGF clinical trials in CNS disease). In the first study, a single patient was treated with murine NGF (mNGF) infusion into the right ventricle [119]. The treatment resulted in a marked transient increase in uptake and binding of ^11^C] -nicotine in the frontal and temporal cortex, a persistent increase in cortical blood flow and a progressive decrease of slow wave EEG activity Tests of verbal episodic memory were also improved whereas other cognitive tests were not. As relevant side effect, weight loss was reported [119]. The results of a second study on ICV mNGF delivery was published in 1998 [120]. The relative positive outcomes found in the first study were confirmed in the two patients treated with higher doses of mNGF. However, the major finding of the study could be considered the occurrence of important and severely limiting side effects: reversible weight loss during the mNGF infusion period and, most importantly, development of back pain symptoms after the beginning of ICV infusion that most probably reflects the NGF-mediated hyper-activation of nociceptive transmission system in the spinal cord. These side effects were considered to outweigh the positive outcomes and lead to discontinuation of ICV infusion-based trials in AD patients.

**Table 2:** Summary of clinical trials with NGF on central nervous system’s diseases

**Disease**	**Study type**	**NGF type and dosage**	**Delivery route**	**Outcome**	**Side effects**	**References**
Alzheimer’s Disease	Single case report.	Mouse NGF. 75 μg/day for three months, total amount: 6.6 mg.	ICV.	Increase of cortical blood flow and brain nicotine uptake. Improvement of verbal episodic memory.	Weight loss.	[119]
	Three patients case report.	Mouse NGF. Two patients:75 μg/day or three months, total amount: 6.6 mg. One patient: 16 μg/day for 2 weeks and 3.4 μg/day for further 10 weeks, total amount: 0.55 mg.	ICV.	Increase of brain nicotine uptake.	Weight loss. Back pain.	[120]
	Phase I clinical trial.	Human NGF genetically engineered into autologous grafted fibroblasts.	Gene therapy.	Improvement in the rate of cognitive decline. Significant increases in cortical 18-fluorodeoxyglucose after treatment, as revealed by PET scans.	Absence of long-term adverse effect in 6 out of 8 patients.	[182]
	Phase I randomized, controlled dose-escalating study to assess the safety and tolerability of CERE-110.	Human NGF genetically engineered into adeno-associated virus vector (CERE-110).	Gene therapy.	Ongoing.	Ongoing.	[183-185]
	Open label, 12 month study on 6 patients.	Human NGF genetically engineered in human retinal cells encapsulated in implantable device	Gene therapy.	The phase I trial was a safety and tolerability study. The implantation and removal of device were safe and well tolerated. Positive neurological outcomes were also found in 2 out of 6 patients.	No NGF-related adverse events were found.	[187,190]
Parkinson’s disease	Single case report.	Mouse NGF. 3.3 mg infused via implanted cannula over 23 days, as support for adrenal medulla graft.	Intra-putaminal.	NGF treatment could prolong the effect of adrenal chromaffin grafts in human PD.	Not reported.	[121]
Optic glioma and advanced optic nerve atrophy.	Five patients case study.	Mouse NGF. 1 mg total over 10 days in daily applications.	Topical (eye).	Improvement in visual evoked potentials (VEP).	Not reported.	[206]
	Single patient case study.	Mouse NGF. 1 mg total over 10 days in daily applications.	Topical (eye).	Reversible improvements of visual function and electrophysiological measurements.	Not reported.	[207]
Hypoxic-ischemic perinatal brain injury	Two patients case study.	Mouse NGF. 0.1 mg/day for 10 days.	ICV.	Improvement in the comatose status, increased alpha/theta ratio in the EEG, reduction of malacic areas and improvement, in right temporal and occipital cortices perfusion.	Not reported.	[123]
	Two patients case study.	Mouse NGF. 0.1 mg/day for 10 days.	ICV.	Improvement in EEG and SPECT parameters. An increase of doublecortin in CSF.	Not reported.	[122]

The ICV NGF infusion has been also pursued in single or small groups of patients in diseases such as Parkinson’s disease (PD) [121] and hypoxic-ischemic perinatal brain injury [122-124]. The rationale for the use of NGF infusion in PD is linked to its supportive role for adrenal medullary cells engrafted in the basal ganglia of PD patients [125,126]. The cell replacement therapy for PD patients by autologous chromaffin adrenal tissue grafting into the caudate nucleus was pursued in the 1980’s [127,128], and based on previous positive indications coming from animal studies on models of PD [129,130]. Overall, these studies lead to consensus over the lack of long-lasting effects, due to lack of specific support to engrafted cells. NGF ICV infusion was then attempted, in light of NGF effects on survival, neurite outgrowth, and functionality of grafts of adrenal chromaffin cells to the basal ganglia. The study [121] reports the case of a 63 year-old patient that underwent autologous graft of adrenal medulla into the putamen, supported by a 23 day ICV infusion of mNGF. During the 13 months follow up the patient had a rapid decrease in rigidity and hyperkinesias that was similar to what observed in previous studies on autologous graft of chromaffin tissue in PD patients. The specific effect of mNGF support to the graft was identified in a slower improvement of motor functions that was extended for 11 months after the graft procedure. Thus NGF treatment could prolong the effect of adrenal chromaffin grafts in human PD [121].

As for the clinical studies performed on children with traumatic brain injury (TBI), the rationale for NGF utilization comes from animal studies, showing that NGF can reduce neurological deficits following brain injury in animals [131], and from the observation that NGF levels in the cerebro-spinal fluid (CSF) of TBI patients have a positive correlation with neurological outcomes [124]. Two studies were attempted with an ICV infusion of mNGF in children with TBI. In the first study [123] two infants aged 8 and 9 months were treated with mNGF infused into the right cerebral ventricle for 10 days starting 30 days after the hypoxic-ischemic brain injury. Very preliminary observations detected an improvement in the comatose status, increased alpha/theta ratio in the EEG, reduction of malacic areas and improvement, in one child only, of the regional cerebral perfusion in right temporal and occipital cortices, as measured by SPECT. A second study [122] in 2 infants aged 8 and 13 months and affected by hypoxic-ischemic brain damage, showed the results of mNGF ICV infusion, starting 4 months after TBI. Again, an improvement in EEG and SPECT parameters was scored with a concomitant increase of doublecortin, a protein expressed by newly formed neurons, in the CSF. These studies, tough limited by a small number of patients, indicated a possible effect of NGF in the treatment of TBI secondary to hypoxic-ischemic brain insult, but did not investigate potential side effects linked to the ICV infusion.

### Skin ulcers

Beside its action as a neurotrophic factor for nerve cells, NGF has been characterized as a regulatory factor for many non-neuronal cell types, expressing NGF receptors [2]. The role of NGF on skin biology is particularly relevant from a clinical perspective. Production and utilization of NGF has been demonstrated in skin cells, as keratinocytes [132-134], and in immune cells that are resident or recruited in epidermal tissue following trauma or inflammation [135-137]. NGF deregulation has been described in diseased skin [137-143]. The effects of NGF on the healthy and diseased skin could be directly exerted via NGF receptors expressed on epidermal and dermal cells, or by NGF influence on PNS skin innervation that is known to regulate skin homeostasis by neuropeptides and neurotransmitter release [144-152]. The possible role of NGF as a therapeutic in skin trauma and/or diseases was investigated in animal models of wound healing [62,140,152,153]. Later on, topical application of NGF has been pursued in several forms of epithelial derangements and skin disease (summarized in Table 3).

**Table 3:** Summary of clinical trials with NGF on skin ulcers

**Disease**	**Study type**	**NGF type and dosage**	**Delivery route**	**Outcome**	**Side effects**	**References**
Diabetic foot ulcers	Three patients case report.	Mouse NGF. 25 μg/day for 4 weeks.	Topical (skin).	Progressive restoration of nerve function and relapse of ulcers within 5–14 weeks since the beginning of treatment.	Not reported.	[154]
Vasculitic ulcers	Eight patients case report.	Mouse NGF. 50 μg/day for 4 weeks.	Topical (skin).	Ulcers healing within 8 weeks in rheumatoid arthritis patients (n=4). Failure of ulcers healing in systemic sclerosis patients (n=4).	Not reported.	[155]
Pressure ulcers	Single patient case study.	Mouse NGF.	Topical (skin).	Ulcer size reduced by 1/3 after 15 days treatment.	Not reported.	[156]
	Randomized, double-blind, placebo-controlled trial.	Mouse NGF.	Topical (skin).	Reduction of ulcer area in the 6 weeks follow-up.	Not reported.	[157]
Lower limb crush syndrome	Single patient case study.	Mouse NGF. 10 μg every eight hours for seven days.	Subcutaneous.	Reduction of overall ischemic area. Reduction of the area undergoing calcaneal escharotomy.	Not reported.	[158]

Topical mNGF has been applied in three diabetic patients affected by on foot ulcers [154]. The treatment induced a local progressive restoration of nerve function and an almost complete relapse of ulcers within 5–14 weeks since the beginning of treatment.

Another clinical study was performed in patients affected by chronic vasculitic ulcers secondary to rheumatoid arthritis (RA) or systemic sclerosis (SSc) [155]. The leg ulcers of the patients with rheumatoid arthritis (n=4) showed a rapid reduction in volume which led, in all cases, to heal within 5–8 weeks. Descriptive variables such as pain, presence of granulation, absence of inflammation also improved in the same period. SSc patients (n=4) were treated on both the hand and leg ulcers. Despite little improvement in inflammatory states and ulcer size, none of the ulcers reached healing after 8 weeks. The authors speculated over the different effects in RA and SSc ulcers as attributable to disease features diversity, especially in the microvascular fibrosis that characterizes SSc and could reduce NGF access to damaged cells [155].

Pressure ulcers have been also treated with topical mNGF and results reported in two separate studies from the same group [156,157]. The first study described a single patient affected by pressure ulcers bilaterally located on the elbows [156]. The right elbow was treated with mNGF and the ulcer was reduced by 1/3 while the left elbow ulcer was substantially unchanged. In the second report, a randomized, double-blind, placebo-controlled trial was described, aimed at investigating the effects of topical treatment with mNGF in patients with severe, non-infected pressure ulcers of the foot [157]. Topical mNGF was applied to 18 patients, while another 18 patients received vehicle treatment only. The average reduction in pressure ulcer area after 6-week follow-up period was statistically significantly greater in the treatment group than in the control group.

Another interesting case report investigated the effect of topical NGF treatment in ischemic skin revascularization [158]. In this case the described effects of NGF as a promoter of vascular-endothelial growth factor (VEGF) and neo-vascularization [159] gave the rationale background for treatment of a child with a severe crush syndrome of the lower left limb with subcutaneous mNGF. The gradual improvement of the ischemic treated area was observed throughout the treatment period, with a significant reduction in size of the overall ischemia and a final outcome identified in a reduction of the area that finally underwent calcaneal escharotomy [158]. It is worth noticing that the pro-angiogenic activity of NGF could be of therapeutic relevance in various types of cancer, where positive correlations between cancer stage/prognosis and tissue NGF levels have been described [160-162]. Thus, targeting the NGF/VEGF interaction system should be also regarded as a potential new strategy for anti-angiogenic therapy against cancer as well as for other angiogenesis-dependent diseases, such as diabetes, and arthritis [162,163].

### Ophthalmology

The use of NGF as a therapeutic in ophthalmology is perhaps the best characterized and developed, among the other possible or yet pursued clinical use (refer to Table 4 for a summary of clinical trials of NGF in ophthalmology). One of the first evidence suggesting a possible role of NGF in the visual system was reported in 1979 by Turner [164] who showed that the retinal cells of goldfish are receptive to the action of NGF. It has been reported that NGF induces modification of pre-synaptic elements in adult visual system [165,166], prevents the shift in ocular dominance distribution of visual cortical neurons and promotes functional recovery of retinal ganglion cells (RGC) after ischemia [167], delays retinal degeneration in rodents with inherited retinopathy [168,169], reduces retinal damages in rabbits with ocular hypertension [170], while injection of antibody against NGF exacerbate the damaging effect on RGC [170]. The first attempt to translate preclinical studies into clinic was based on the described presence of NGF and NGF receptors on corneal cells and structure [171-175], suggesting a possible trophic influence of the neurotrophin on the ocular surface.

**Table 4:** Summary of clinical trials with NGF in ophthalmology

**Disease**	**Study type**	**NGF type and dosage**	**Delivery route**	**Outcome**	**Side effects**	**References**
Neurotrophic keratitis	Twelve patients case report.	Mouse NGF. Several daily applications of a 200 μg/ml solution for 6 weeks.	Topical (eye).	Healing of all of the ulcers, improved corneal sensitivity and integrity and improved visual acuity.	Not reported.	[172]
	Prospective, noncomparative, interventional case series; 43 patients.	Mouse NGF. Several daily applications of a 200 μg/ml solution until ulcer healing.	Topical (eye).	Complete resolution of the epithelial defect between 12 days and 6 weeks of treatment. Improvement of corneal sensitivity and visual acuity.	Hyperemia and ocular and periocular pain.	[176]
	Observational study on 11 patients.	Mouse NGF. Several daily applications of a 200 μg/ml solution until ulcer healing.	Topical (eye).	Ulcer healing between 9 and 43 days after initiation of treatment. No development of systemic anti-NGF antibodies in a follow-up time of 72 months.	Mild and transient conjunctival hyperemia and photophobia.	[177]
Glaucoma	Three patients case report.	Mouse NGF. Four daily applications of a 200 μg/ml solution for 3 months.	Topical (eye).	Progressive improvement in the functionality of the inner retinal layer and in the parameters of the post-retinal neural conduction and visual acuity, maintained for 3 months after discontinuation of treatment.	Local burning during the first week of treatment in a single patient.	[178]
Bilateral age-related macular degeneration (retinopathy)	Single case study.	Mouse NGF. Three times daily applications of 200 μg/ml solution for 2 separate periods of 1 year and 5 years in the right eye.	Topical (eye).	Improvement in visual acuity and in the amplitude of the ERG.	Slight burning at the time of application of eye drops during the first month of treatment.	[179]

In the first published study about the application of NGF-eyedrops [172], severe corneal ulcers associated with anesthesia (corneal neurotrophic keratitis) were treated with purified mNGF. The results reported a rapid healing of all of the ulcers, improved corneal sensitivity and integrity and improved visual acuity. Very similar results were reported in a following study, on neurotrophic keratitis non-responsive to conventional treatments [176]. The follow-up lasted for a period ranging between 3 and 32 months after initiation of treatment and the study shows that all patients achieved complete healing of the corneal defect within a period between 12 days and 6 weeks after initiation of treatment. The study also showed that the occurrence of side effects, described as hyperemia and moderate pain in the eye and periocular area, were well tolerated and limited to the time necessary for the remission of corneal keratitis.

A study published in 2007 evaluated the effect of topical treatment with mNGF on eyes from 11 patients with neurotrophic keratopathy [177], considering in particular the possible occurrence of unpleasant side effects and the development of systemic anti-NGF as a result of the treatment protocol. All patients had healing of corneal ulcers between 9 and 43 days after initiation of treatment. The study revealed that the ocular discomfort lasted less than an hour after the instillation of eye drops and that any painful sensation disappeared, even when NGF treatments were continued after the healing of corneal ulcers. None of the patients developed systemic symptoms during treatment or during follow-up. The presence of antibodies against the mNGF in the blood was negative for all of the treated patients during the therapy and for a period of follow-up up to 72 months.

After the first approach on ocular surface pathologies, mNGF was used in clinical studies addressing the posterior segment of the eye. In a study published in 2009, the application of topical mNGF, was evaluated in three patients with advanced glaucoma, with imminent risk of loss of visual function [178]. The reported results were: progressive improvement in the functionality of the inner retinal layer and in the parameters of the post-retinal neural conduction, evident during the treatment period and maintained even 3 months after discontinuation of treatment; visual acuity improved significantly in all patients where it remained unchanged for the 3-month follow-up. The study also showed the substantial absence of side effects, except for the development of local burning during the first week of treatment in a single patient.

Another case report was about the use of topical mNGF to treat a patient suffering from bilateral macular degeneration (AMD) [179]. The treatment was continued for 6 years virtually uninterrupted. Checks were made on a quarterly basis and showed a clinical (improvement of visual acuity) and electrofunctional (increasing the amplitude of the ERG) improvements in the right eye, correlated with treatment. The only side effect noted was a sensation of slight burning at the time of application of eye drops during the first month of treatment.

It is worth noticing that in none of the cited studies systemic side effects attributable to the biological action of NGF itself were reported. In particular, the ocular NGF seems not to give rise to systemic effects on the perception of pain (myalgias, hyperalgesia) reported in clinical trials with systemic [87] or intra-cerebroventricular [120] administration.

## Novel delivery routes

Though applied to a wide spectrum of neurological and non-neurological diseases, clinical utilization of NGF, especially when systemically administered, remains hampered by important adverse events, such as those derived from the effects of NGF on pain system. Moreover, the achievement of pharmacological concentrations in therapeutic relevant targets without affecting non-target areas represents a further delivery challenge.

A potential approach to overcome such limitations is represented by gene therapy. Preclinical data obtained in rodents and primates indicated that ex vivo gene therapy targeted at BFC neurons were effective in improving experimentally induced cholinergic deficits [180,181]. A phase I clinical trial has been performed on 8 AD’s patients, in which autologous fibroblasts were engineered to produce and secrete human NGF (hNGF) and implanted into the BFC (Table 2) [182]. Positive outcomes in behavioral scales were scored in two out of six patients that survived cell implant neurosurgery in a period ranging from six to eighteen months after implant, associated with improvement in PET scans. A second phase I trial based on in vivo NGF gene delivery, by adeno-associated virus vector (CERE-110) [183] has been set-up in 2004 (Table 2) [184]. The phase I study was a dose-escalating study to assess the safety and tolerability of CERE-110 in subjects with mild to moderate AD’s. CERE-110 has passed phase I clinical testing and a multicenter phase II clinical trial has commenced [185]. In addition to the CERE-110 trial, a new, cell-based in vivo delivery system has been developed and a Phase I trial has been registered by Karolinska Institute in Sweden on AD’s patients (Table 2) [186]. This delivery system is based on human retinal pigment epithelial cell line, engineered to secrete hNGF and encapsulated within a polymer membrane that is part of an implantable catheter-like device [187], and has been demonstrated to effectively prevent the loss of cholinergic neurons after fimbria transection in rats [188,189]. The results of the first safety and tolerability study based on such system have been recently published [187,190], demonstrating that surgical implantation and removal of devices containing NGF-secreting cells in the basal forebrain of AD patients are feasible, well tolerated and relatively safe, and that they do not generate NGF-related adverse events [190]. Though the system remains to be optimized in terms of long-term function of implanted cells and in the achievement of positive neurological outcomes (actually limited to 2 out of 6 patients), it seems to present advantages over other gene delivery approaches [182], since it allows the safe removal of engineered cells as well as their immune isolation from host tissues, thus protecting the allogeneic transplanted cells from host immune system rejection.

The olfactory pathway is a promising, non-invasive route for drug delivery to the brain, which has potential for the treatment of neurodegenerative diseases [191-193]. The BBB represents one of the major obstacles in drug development for brain diseases and many studies have focused on the possibility of circumventing the BBB for the direct central delivery of macromolecules to the central nervous system by utilizing the potential direct transport pathway from nose to brain via the olfactory region [194-196]. The intranasal delivery of NGF to the brain could indeed represent a non-invasive and safe route to achieve relevant therapeutic concentration of NGF in selected brain areas, without eliciting undesired and adverse side effects. The characterization of pharmacokinetic for intranasal delivery of NGF [197,198] revealed that intranasal NGF rapidly spread through brain tissue without significant increasing NGF concentrations in the CSF and in the blood. Studies on animal models of AD, revealed that intranasal NGF, while exerting specific therapeutic actions on the affected cholinergic system, did not provide trophic support to sympathetic ganglia, nor did it induced the over expression of nociception neuromodulators, such as sensory neuropeptides, known to be under NGF control [197-199]. Recent studies published by the same group, characterized a form of hNGF mutated at residue R100, testing it both in vitro and in vivo, by intranasal delivery, in an animal model of AD [199,200]. Such a mutated hNGF retains the neurotrophic potential of the native NGF, without eliciting pain-related response. It would be of interest whether such a “painless” NGF variant can be validated in preclinical and hopefully future clinical trials for neurodegenerative diseases [199].

Another actually investigated route that seems to be able to deliver NGF to the brain in a safe and effective manner is the topical administration of NGF on ocular surface [201]. Animal studies have demonstrated that NGF applied on the ocular surface can reach central cholinergic neurons, which are affected in AD [201]. Moreover, ocular NGF is able to activate c-fos in several areas of the limbic system in a time-dependent manner [202] and to enhance the distribution of Ki67positive cells also expressing p75^NTR^ in the proliferating layer of the sub-ventricular zone, indicating that ocular NGF can activate the machinery regulating the proliferation and maturation of neuronal precursor in the brain [203]. Compared to intranasal delivery, the intraocular one appears to be less characterized, in terms of mechanisms and anatomical route for brain delivery [204]. The available data indicate that the ocular delivered NGF does not induce systemic side effects related to the systemic NGF administration, even when this is repeated for rather long time periods [172,176,177,179], being able to target selected brain areas [201-203]. In a recent study on a mouse model of AD the intranasal and ocular delivery routes for hNGF administration were compared for their relative diffusion in the systemic compartment in concentration that could elicit a pain response [205]. The authors reported that intra-nasally applied hNGF was safer when compared to intra-ocular one. It should however be taken into consideration and tested whether there is a possible different biological activity of mNGF used in the majority of the ophthalmology clinical trials [172,176,177,179] versus the rhNGF used in the cited comparative study [205].

To date two reports, both from the same group, investigated the clinical effects of topical ocular NGF on brain structures lying behind the retina (Table 2) [206,207]. In one study [206] five pediatric patients with optic gliomas (OGs) and advanced optic nerve atrophy were assessed before and after a single 10 day course of 1 mg (total) mNGF topical administration by clinical evaluation, visual evoked potentials (VEPs), and brain magnetic resonance imaging (MRI). While not affecting tumor size, the topical mNGF improved VEPs suggesting a visual rescuing mechanism exerted by mNGF on the residual viable optic pathways. In a further study [207] a single adult patient with OG and long-standing optic nerve atrophy was treated with mNGF and the follow-up was performed by clinical, neuroradiologic, and electrophysiological tests (electroretinogram and VEPs) at the end of each treatment and 30 and 60 days later. Repeated subjective and objective improvement of visual function was recorded after mNGF treatment, which tended to deteriorate toward baseline values 60 days from the end of each mNGF treatment. Interestingly, no ocular or systemic side effects were observed throughout treatment [207].

## Conclusions

Soon after its discovery, in the middle of the twentieth century, it became clear that NGF had great pharmacological potentialities, for the treatment of major central neurodegenerative diseases and of peripheral neuropathies. After preclinical characterization and clinical trials have been performed by treating AD, Parkinson’s, and diabetic patients (Figure 2, Tables 1 and 2), severe limitations in the clinical use of NGF emerged, coming from its physiological action on the sensory and autonomic systems and from the high pharmacological doses needed to obtain disease improvements. Despite the discouraging results coming from trials mainly performed across the 1990’s, the translational research on NGF was not stopped, widening the spectrum of diseases that could benefit from NGF-based therapy and investigating new delivery strategies, aimed at maximizing positive outcomes and limiting or fully circumventing the deleterious side effects described in earlier clinical trials. Today we know that epithelial derangements based on poor neurotrophism could be safely treated with topical NGF, while a wide spectrum of CNS and PNS diseases will probably benefit from NGF therapy, once intranasal or gene delivery systems will be finally set-up and fully translated into clinical practice. A further challenge, in conclusion, is represented by the increasing knowledge on the role of NGF in immune system regulation, opening a promising field for development of innovative NGF-based therapies in the care of, in example, chronic inflammatory or autoimmune diseases, and a novel and challenging aspect in the NGF saga.

## Abbreviations

AD: Alzheimer’s disease; BBB: Blood–brain barrier; BFC: Basal forebrain complex; CGRP: Calcitonin gene-related peptide; CIPN: Chemotherapy-induced peripheral neurotoxicity; CNS: Central nervous system; CSF: Cerebro-spinal fluid; DRG: Dorsal root ganglion; ERK: Extracellular signal-regulated kinase; HIV: Human immunodeficiency virus; hNGF: Human NGF; ICV: Intra-cerebro-ventricular; MAPK: Mitogen activated protein kinase; mNGF: Murine NGF; MRI: Magnetic resonance imaging; NF-κB: Nuclear factor kappa-light-chain-enhancer of activated B cells; NGF: Nerve growth factor; OG: Optic glioma; p75^NTR^: p75 pan-neurotrophin receptor; PD: Parkinson’s disease; PI3K: Phosphatidylinositol 3-kinase; PLC: Phospholipase C; PNS: Peripheral nervous system; RA: Rheumatoid arthritis; RGC: Retinal ganglion cell; rhNGF: Recombinant human NGF; SP: Substance P; SSc: Systemic sclerosis; TBI: Traumatic brain injury; TH: Tyrosine hydroxylase; TrkA: Tropomyosin kinase receptor A; VEGF: Vascular-endothelial growth factor; VEPs: Visual evoked potentials.

## Competing interests

The authors declare that they have no competing interests.

## Authors’ contributions

LA conceived, drafted and reviewed the manuscript. MLR drafted and reviewed the manuscript. PB drafted and reviewed the manuscript. LM conceived, drafted and reviewed the manuscript. All authors read and approved the final manuscript.
